# Drug-induced fall risk in older patients: A pharmacovigilance study of FDA adverse event reporting system database

**DOI:** 10.3389/fphar.2022.1044744

**Published:** 2022-11-29

**Authors:** Shuang Zhou, Boying Jia, Jiahe Kong, Xiaolin Zhang, Lili Lei, Zhenhui Tao, Lingyue Ma, Qian Xiang, Ying Zhou, Yimin Cui

**Affiliations:** ^1^ Department of Pharmacy, Peking University First Hospital, Beijing, China; ^2^ Department of Pharmacy, The First Hospital of Tsinghua University, Beijing, China; ^3^ China Pharmaceutical University, Basic Medicine and Clinical Pharmacy, Nanjing, Jiangsu, China; ^4^ Department of Geriatrics, Peking University First Hospital, Beijing, China; ^5^ Department of Nursing, Peking University First Hospital, Beijing, China; ^6^ Institute of Clinical Pharmacology, Peking University, Beijing, China

**Keywords:** older patients, pharmacovigilance, risk of drug-induced falls, FARES, ADR

## Abstract

**Objectives:** As fall events and injuries have become a growing public health problem in older patients and the causes of falls are complex, there is an emerging need to identify the risk of drug-induced falls.

**Methods:** To mine and analyze the risk signals of drug-induced falls in older patients to provide evidence for drug safety. The FDA Adverse Event Reporting System was used to collect drug-induced fall events among older patients. Disproportionality analyses of odds ratio (ROR) and proportional reported ratio were performed to detect the adverse effects signal.

**Results:** A total of 208,849 reports (34,840 fall events and 1,898 drugs) were considered. The average age of the included patients was 76.95 ± 7.60 years, and there were more females (64.47%) than males. A total of 258 drugs with positive signals were detected to be associated with drug-induced fall incidence in older patients. The neurological drugs (104, 44.1%) with the largest number of positive detected signals mainly included antipsychotics, antidepressants, antiparkinsonian drugs, central nervous system drugs, anticonvulsants and hypnotic sedatives. Other systems mainly included the circulatory system (25, 10.6%), digestive system (15, 6.4%), and motor system (12, 5.1%).

**Conclusion:** Many drugs were associated with a high risk of falls in older patients. The drug is one of the critical and preventable factors for fall control, and the risk level of drug-induced falls should be considered to optimize drug therapy in clinical practice.

## 1 Introduction

Falls among older patients have become a growing public health problem. The total number of deaths caused by falls and disability-adjusted life years has been steadily increasing worldwide since 1990, with deaths nearly doubling by 2017 (James et al., 2020). Falls are particularly problematic among older individuals. The World Health Organization reported that the annual rate of falls was 28–35% among older individuals in the community aged ≥65 years and 32–42% of those aged ≥70. In addition, the fall rate was higher among older individuals living in nursing homes (Yoshida, 2007).

In addition, falls tend to cause more harm and severe consequences in older individuals than in young individuals. A cross-sectional study of 374,972 patients in the United States showed that the age-adjusted mortality rate caused by falls increased from 29.40 per 100,000 in 1999 to 63.27 per 100,000 in 2017 (Shaver et al., 2021). The incidence of falls is higher in aging countries. For example, it has been reported that the proportion of moderate to severe injuries caused by falls was 37.21%, while the proportion of injuries by falls requiring hospitalization was 22.49% (Lu et al., 2021), representing the leading cause of injury-related death among people ≥65 years old in China (Karani et al., 2016; Lu et al., 2021). Falls are responsible for the limited activity, limited function, disability, and even death in older individuals, which seriously affects the health and quality of life of such people (Friedman et al., 2002) and consumes many medical resources (Wang et al., 2010; Gill et al., 2013).

Falls among older individuals result from a multifactorial interaction among limb weakness, unsteady gait, balance disturbance, polypharmacy, history of falls, confusion, age, sex, visual deficits, cognitive decline, especially in attention and executive dysfunction, and environmental and other factors. Drugs are one of these risk factors that cannot be ignored (Ambrose et al., 2013). A cross-sectional study based on U.S. Medicare data showed that the incidence of falls among drug users was as high as 10.3%, compared to 5.42% in people with no history of drug use (Watanabe, 2016). The percentage of people treated with at least one prescription that increased the incidence of falling increased from 57% in 1999 to 94% in 2017 (Gillespie et al., 2012; Shaver et al., 2021). In addition, polypharmacy is an important factor that cannot be ignored. A cross-sectional study evaluated 262 geriatric outpatients showed polypharmacy rather than number of comorbidities was associated with fall risk (Kojima et al., 2011). Taking multiple medications is considered a risk factor for falls through the adverse effects of drug-disease or drug-drug interactions. Falls studies have determined that taking ≥4 drugs is associated with an increased incidence of falls, recurrent falls, and injurious falls (Zia et al., 2015). The Health, Aging and Body Composition Study included 1764 community-dwelling adults demonstrated polypharmacy, particularly combined with fall risk increasing drugs use, was associated with increased risk for treated fall injuries from inpatient and outpatient settings (Xue et al., 2021). Therefore, the cautious use of fall risk increasing drugs is critical for reducing the occurrence of falls.

To effectively reduce the incidence of falls and their hazards, the first necessary step is to assess the risk of falls accurately. At present, several scales have been used for the comprehensive assessment of fall risk (Park, 2018). However, the assessment of drug-induced risk is too simple and not wide, thorough enough. For example, the part about drug-induced fall in the Morse scale (Morse et al., 1989), which is currently and widely used, only focuses on whether intravenous fluid or heparin sodium is used (none = 0 score, yes = 20 scores); the drug-related content in the STRATIFY fall assessment scale (Oliver et al., 1997) is entirely missing, while the drug-related part in the Hendrich II Fall Risk Scale (Hendrich et al., 1995) only includes antiepileptic drugs (no = 0, yes = 2) and benzodiazepines (no = 0, yes = 1).

According to previously published studies, drugs such as hypnotics, antipsychotics, antidepressants, and opioids are associated with an increased risk of causing falls (Hart et al., 2020; Osman et al., 2022; Zidrou et al., 2022). We also noticed that the available data were derived from pre-marketing clinical trials and observational studies which have limited the population, diseases and drugs used, respectively. There is a serious lack of more simple size studies and relevant data on the adverse effects of falls in the real world. According to the above limitations and requirement, spontaneous reporting studies seem worthwhile. FAERS is the world’s largest adverse event self-reporting database, which helps health care workers and the public learn about the postmarketing safety information of drugs and is made available through the FDA Safety Information and Adverse Event Reporting Program (MedWatch) (Sakaeda et al., 2013). Although it is particularly important to identify drugs that could induce falls, to the best of our knowledge, no studies have identified a list of drugs that might increase the risk of falls based on the FAERS.

The purpose of this study was to analyze the drugs related to the adverse effects of falls in the FAERS and to mine the drug-induced risk signals that may increase the risk of falls in older individuals to identify the risk levels that may increase the risk of falls, provide evidence for the selection of clinical drugs and the reduction of fall events, and finally improve the safety of drug use among older individuals.

## 2 Methods

### 2.1 Data source

Data were collected from the FAERS database from 1 January 2004, to 31 December 2020. The Open Vigil 2.1-MedDRA tool (http://h2876314.stratoserver.net:8080/OV21d2/search/) was used to retrieve and extract relevant data (Sakaeda et al., 2013). The inclusion criteria were as follows: fall-related ADEs in patients aged ≥65 (Preferred Term: fall, Minimum age of patient: 65). The prescription drugs, biosimilars, and over-the-counter drugs were included. Vaccines, dietary supplements and the cases which were not reported were excluded.

### 2.2 Data cleaning

The duplicate entry is considered if the patient ID, reporting date, and drug used are all the same. One of the duplicate entries will be kept and the rest will be deleted. If multiple entries were reported for the same drug with the same patient ID, we retained the last fall event for statistical analysis. One entry was made for each unique primary identifier in the data, and the preferred term for all adverse effects reported for that primary identifier was retained. For all the included fall-related reports, the standardized name of the drug referred to Micromedex, and the drugs with the same standardized names were merged.

### 2.3 Statistical analysis

Signal detection was performed using the ratio of reported odds (ROR) (Sakaeda et al., 2013) and proportional reported ratio (PRR) (Böhm et al., 2016) of the disproportionality method, which is based on a four-grid table to mine potential ADE signals by comparing the proportion of targeted events of the targeted drug with the proportion of targeted events of all other drugs. Signal generation standard of ROR: number of reports (a value) ≥ 3 with the lower limit of 95% Confidence Interval (CI) of the ROR value >1, which indicates generated signal. Signal generation standard of PRR: number of reports (a value) ≥ 3 with PRR value ≥2 and variance (**χ2**)≥4, which indicated generated signal. The selected signal met the criteria of both methods, which suggested a potential correlation between the drug and the event. The enumeration data were expressed as the number of cases and the composition ratio. SPSS version 26.0 software (IBM, United States) and Microsoft Excel 2019 software were used for statistical analysis. SAS JMP Statistical (JMP) software v13.0 was used to draw graphs of signal values.

## 3 Results

### 3.1 Descriptive analysis

**FIGURE 1:**
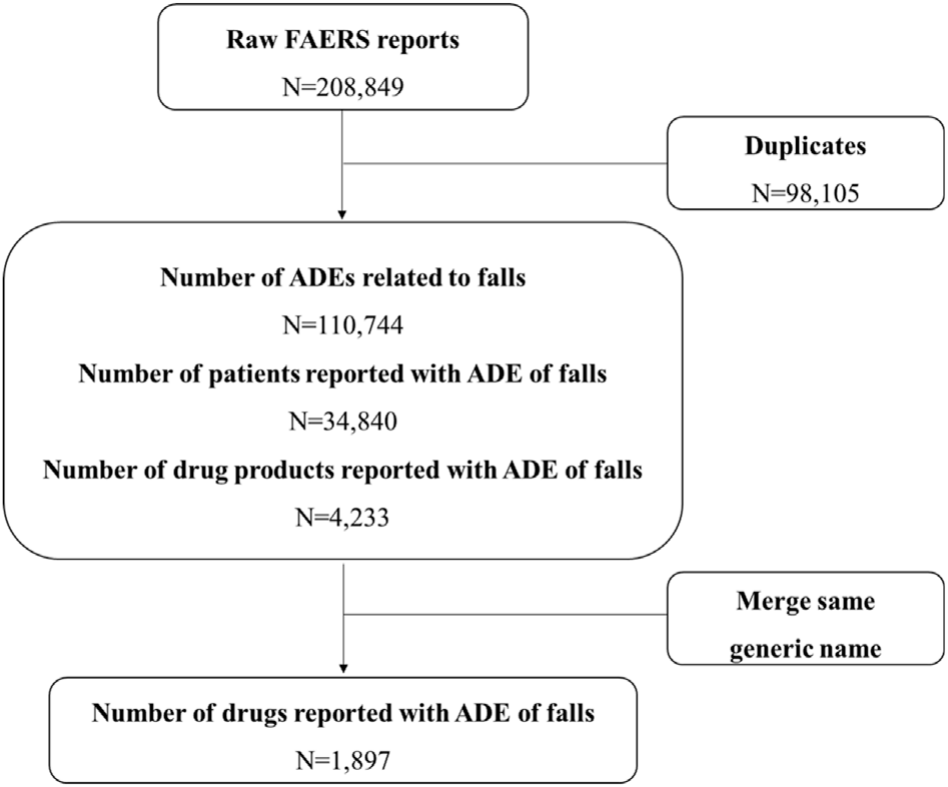
Flow chart for identification of fall reports of suspected adverse events FAERS, FDA Adverse Event Reporting System; ADEs, Adverse Drug Events.

The OpenVigil2.1 database for 68 quarters of data from the first quarter of 2004 to the fourth quarter of 2020 was searched, and a total of 208,849 items were obtained. After removing duplicate information and incomplete data, a total of 110,744 items with complete fall report information were collected, including 34,840 fall events. The data cleaning process is shown in Figure 1.

**FIGURE 2:**
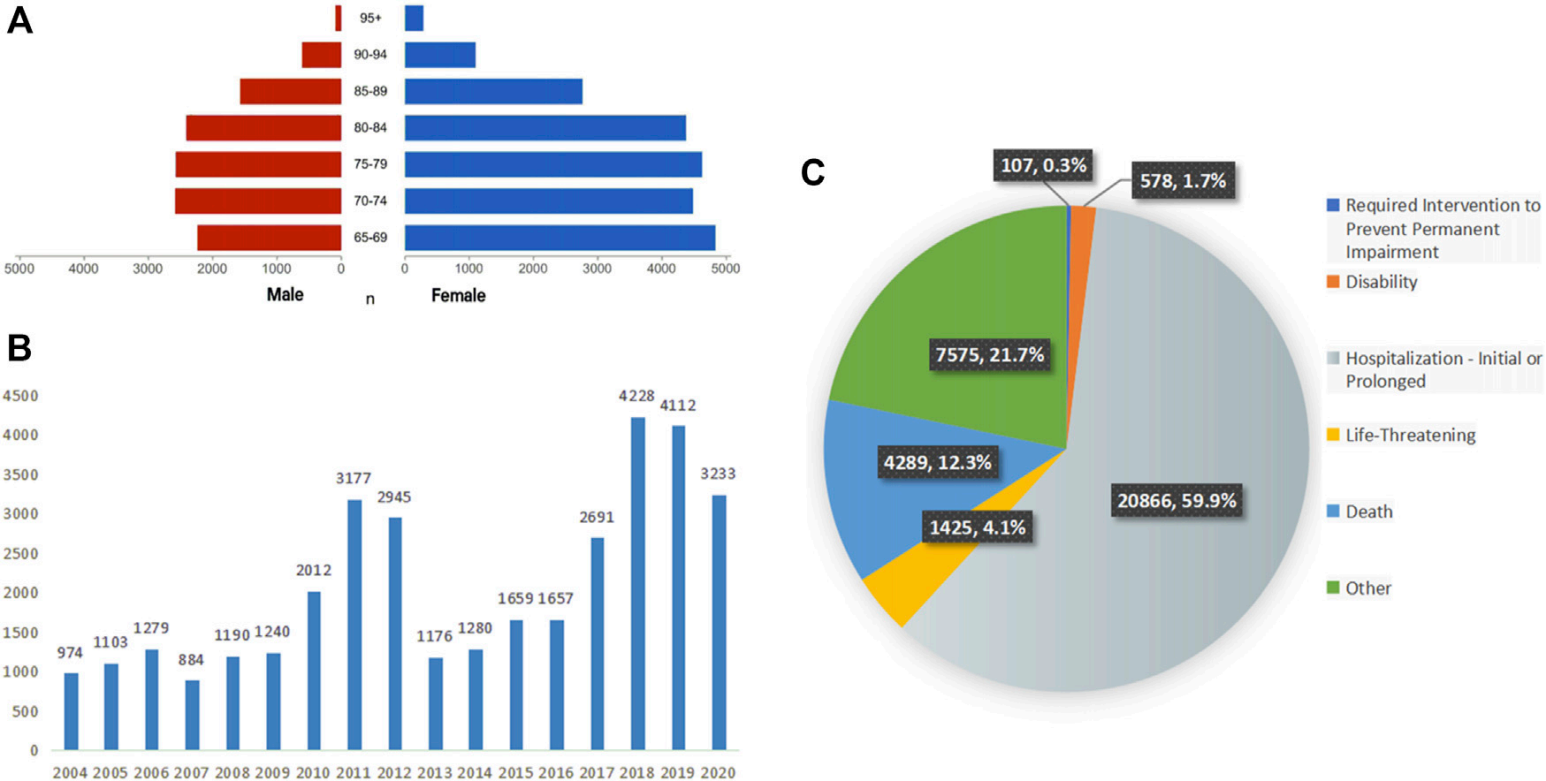
Characteristics of patients and ADEs reports included in the analysis **(A)** the age and sex distribution of included patients, **(B)** the number of ADEs reported per year, **(C)** the outcomes of included patients.

A total of 34,840 patients were enrolled in the analysis, with an average age of 76.95 ± 7.60 years old; there were more women (22,461 cases, 64.5%) than men (12,083 cases, 34.7%). The age and sex distribution of the patients are shown in Figure 2A.

Since the establishment of the database in 2004, ADEs related to falls have been reported every year. During the 17-year period, the number of reported cases showed fluctuating growth, with notable peaks in 2011 and 2012 and the highest peak in 2018. The annual statistics of reported cases are shown in Figure 2B.

A total of 34,840 cases of falls were reported from a total of 92 countries and regions, among which the top five countries in terms of the number of reports were the United States (18,921 cases, 54.5%), Canada (2,380 cases, 6.9%), and the United Kingdom (2,219 cases). Times, 6.4%), France (1,930 cases, 5.6%), and Japan (1,823 cases, 5.3%).

According to the setting of multiple-choice outcomes in the FAERS database, we selected the most severe outcome as the final outcome. Among these, hospitalization or prolongation of hospitalization accounted for the largest proportion, with a total of 20,866 cases, accounting for 59.9%. A total of 4,289 patients died, accounting for 12.3%. The detailed distribution is shown in Figure 2C.

### 3.2 Disproportionality analysis

**FIGURE 3:**
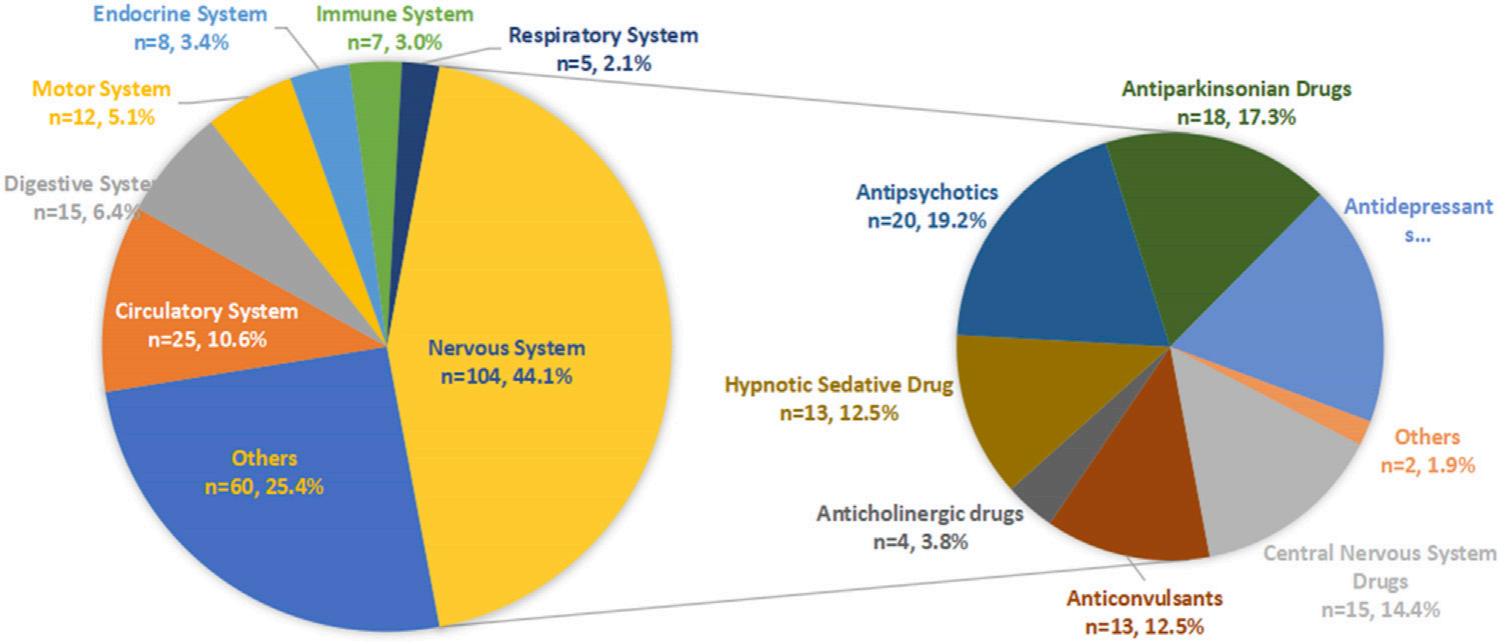
The number of drugs with positive signals detected for each physiological system.

**FIGURE 4:**
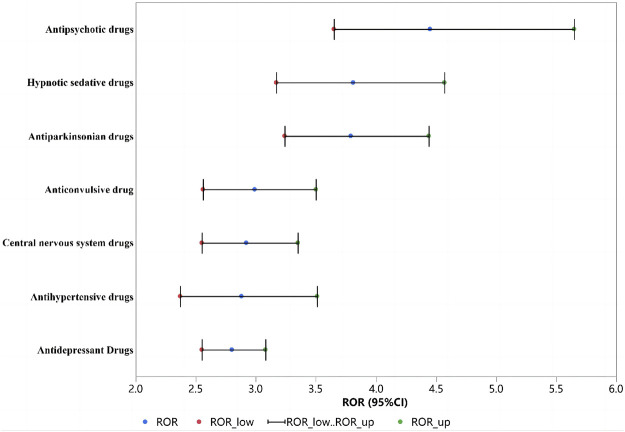
ROR for fall of each physiological system.

After detection of the risk signal, a total of 258 drugs with positive signals for drug-induced falls in the older patients were detected, including 22 compound preparations and the remaining 236 single-drug preparations. In single drug preparations, the number of positive signal drugs found in each physiological system was as follows: nervous system (104, 44.1%), circulatory system (25, 10.6%), digestive system (15, 6.4%), motor system (12, 5.1%), endocrine system (8, 3.4%), immune system (7, 3.0%) and respiratory system (5, 2.1%). Other systems mainly included pain relievers (6, 2.5%), antihistamines (6, 2.5%), nutritional supplements (4, 2.1%), blood regulators, and antitumor drugs (4, 2.1%). The neurological drugs with the largest number of positive detected signals mainly included antipsychotics (20/104, 19.2%), antidepressants (19/104, 18.3%), antiparkinsonian drugs (18/104, 17.3%), central nervous system drugs (15/104, 14.4%), anticonvulsants (13/104, 12.5%) and hypnotic sedatives (13/104, 12.5%). The compositions of the drugs with positive signals detected are shown in Figure 3, and the signal values of each drug class are showed in Figure 4.

#### 3.2.1 Antipsychotic drugs

**FIGURE 5:**
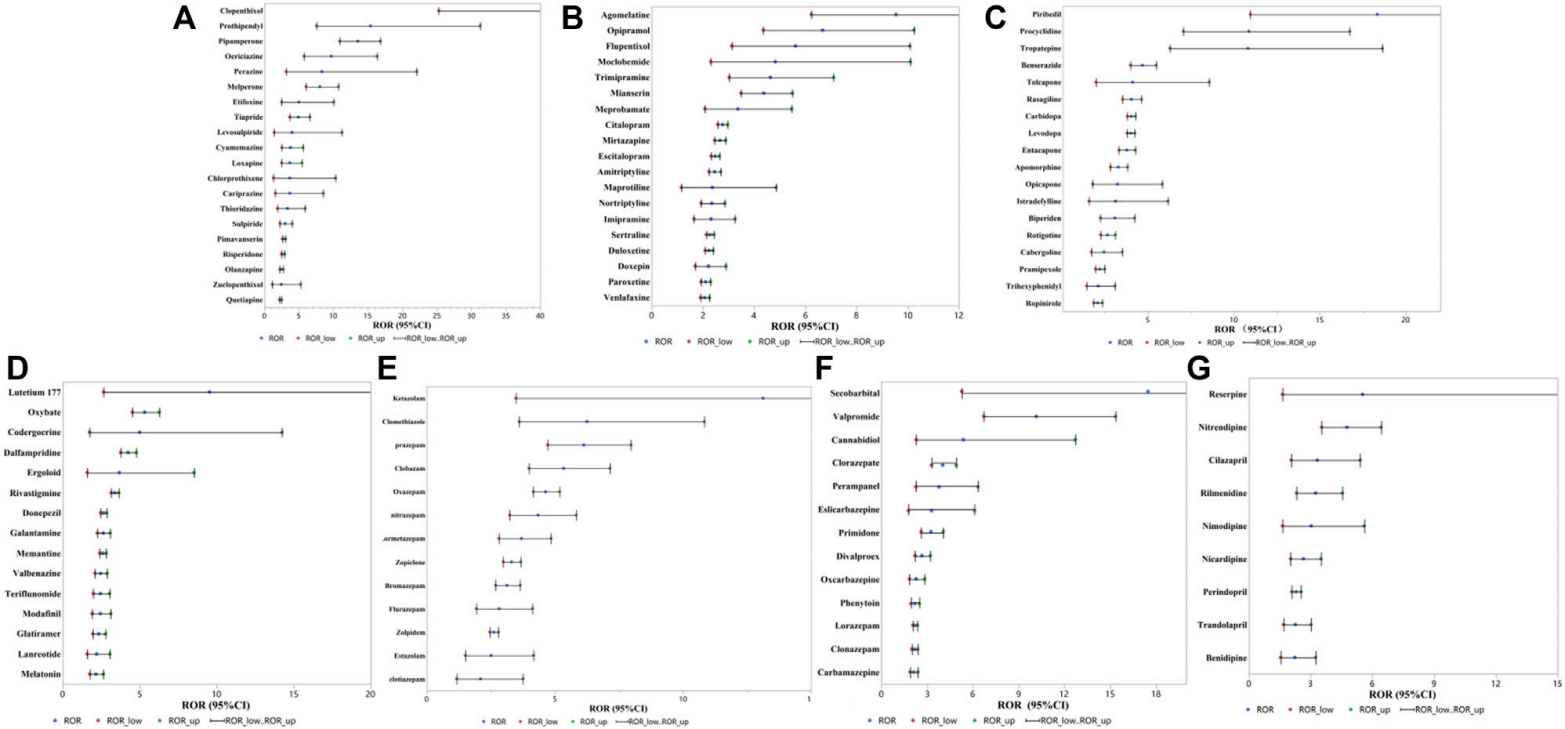
RORs for fall with positive signals detected drugs **(A)** antipsychotic drugs, **(B)** antidepressant drugs, **(C)** antiparkinsonian drugs, **(D)** central nervous system drugs, **(E)** hypnotic sedative drugs, **(F)** anticonvulsive drug, **(G)** antihypertensive drugs.

Among the drugs with positive signals detected, there were 20 antipsychotic drugs. After summarizing according to the random-effects model, the ROR was 4.54 (95% CI, 3.65–5.65). According to the ROR signal intensity, the top three drugs were clopenthixol [ROR (95% CI), 210 (25.28–1744.44), PRR (χ^2^), 30.85 (148.88)], prothionate [ROR (95% CI), 15.4 (7.57–31.3), PRR (χ^2^), 11 (92.81)], pipampirone [ROR (95% CI), 13.55 (10.92–16.83), PRR (χ^2^), 10.0 5 (944.6)]. Other antipsychotic drugs that detected positive signals were periciazine, perazine, melperone, etifoxine, tiapride, levosulpiride, cyamemazine, loxapine, cariprazine, chlorprothixene, thioridazine, sulpiride, pimavanserin, risperidone, olanzapine, zuclopenthixol acetate, and quetiapine fumarate. The signal values of all drugs are shown in Supplementary Table S1 and Figure 5A.

#### 3.2.2 Antidepressant drugs

Among the drugs with positive signals detected, there were 19 antidepressant drugs. After summarizing according to the random-effects model, the ROR was 2.80 (95% CI, 2.55–3.08). According to the ROR signal intensity, the top three drugs were agomelatine [ROR (95% CI), 9.54 (6.23–14.61), PRR (χ^2^), 7.71 (155.44)], opipramol [ROR (95% CI), 6.68 (4.35–10.25), PRR (χ^2^), 5.77 (96.52)], flupentixol [ROR (95% CI), 5.61 (3.12–10.09), PRR (χ^2^), 4.97 (38.51)]. Other antidepressant agents that detected positive signals were moclobemide, trimipramine maleate, mianserin, meprobamate, citalopram, mirtazapine, escitalopram oxalate, amitriptyline, maprotiline, nortriptyline, imipramine, sertraline, duloxetine, doxepin, paroxetine, and venlafaxine. The signal values of all drugs are shown in Supplementary Table S1 and Figure 5B.

#### 3.2.3 Antiparkinsonian drugs

Based on the drugs with positive signals detected, there were 18 antiparkinsonian drugs. After summarizing according to the random-effects model, the ROR was 3.79 (95% CI, 3.79–4.44). According to the ROR signal intensity, the top three drugs were piribedil [ROR (95% CI), 18.34 (10.94–30.72), PRR (χ^2^), 12.37 (225.02)], procyclidine [ROR (95% CI), 10.86 (7.05–16.73), PRR (χ^2^), 8.53 (176.82)], and tropatepine hydrochloride [ROR (95% CI), 10.82 (6.28–18.64), PRR (χ^2^), 8.5 (108.11)]. Other antiparkinsonian agents that detected positive signals were benserazide, tolcapone, rasagiline, carbidopa, levodopa, entacapone, apomorphine hydrochloride, opicapone, istradefylline, biperiden hydrochloride, rotigotine, cabergoline, pramipexole, trihexyphenidyl hydrochloride and ropinirole hydrochloride, and the other 2 compound preparations were carbidopa/levodopa and carbidopa/levodopa/entacapone. The signal values of all drugs are shown in Supplementary Table S1 and Figure 5C.

#### 3.2.4 Central nervous system drugs

Based on the drugs with positive signals detected, there were 15 central nervous system drugs. After summarizing according to the random-effects model, the ROR was 2.92 (95% CI, 2.55–3.35). According to the signal intensity of ROR, the top three drugs were lutetium 177 [ROR (95% CI), 9.54 (2.66–34.21), PRR (χ^2^), 7.71 (11.78)], sodium oxybate [ROR (95% CI), 5.33 (4.52–6.28), PRR (χ^2^), 4.76 (501.42)], codergocrine [ROR (95% CI), 4.99 (1.75–14.25), PRR (χ^2^), 4.49 (7.88)]. Other central nervous system drugs that detected positive signals were dalfampridine, ergoloid, rivastigmine, donepezil, galantamine, memantine, valbenazine, teriflunomide, modafinil, glatiramer, lanreotide, melatonin and compound preparation: dextroamphetamine/amphetamine. The signal values of all drugs are shown in Supplementary Table S1 and Figure 5D.

#### 3.2.5 Hypnotic sedative drugs

Based on the drugs with positive signals detected, there were 13 hypnotic sedative drugs. After summarizing according to the random-effects model, the ROR was 3.81 (95% CI, 3.17–4.57). According to the ROR signal intensity, the top three drugs were ketazolam [ROR (95% CI), 13.12 (3.48–49.47), PRR (χ^2^), 9.81 (16.2)], clomethiazole [ROR (95% CI), 6.25 (3.6–10.82), PRR (χ^2^), 5.45 (51.63)], and prazepam [ROR (95% CI), 6.13 (4.72–7.97), PRR (χ^2^), 5.36 (236.54)]. Other hypnotic sedative drugs that detected positive signals were clobazam, oxazepam, nitrazepam, lormetazepam, zopiclone, bromazepam, flurazepam, zolpidem, estazolam and clotiazepam. The signal values of all drugs are shown in Supplementary Table S1 and Figure 5E.

#### 3.2.6 Anticonvulsive drug

Based on the drugs with positive signals detected, there were 13 anticonvulsants. After summarizing according to the random-effects model, the ROR was 2.99 (95% CI, 2.56–3.50). According to the signal intensity of ROR, the top three drugs were secobarbital [ROR (95% CI), 17.49 (5.26–58.11), PRR (χ^2^), 11.99 (30.93)], valpromide [ROR (95% CI), 10.15 (6.71–15.35), PRR (χ^2^), 8.09 (178.19)], cannabidiol [ROR (95% CI), 5.38 (2.27–12.71), PRR (χ^2^), 4.79 (14.86)]. Other anticonvulsant drugs that detected positive signals were clorazepate, perampanel, eslicarbazepine, primidone, divalproex, oxcarbazepine, lorazepam, phenytoin, clonazepam and carbamazepine. The signal values of all drugs are shown in Supplementary Table S1 and Figure 5F.

#### 3.2.7 Antihypertensive drugs

Based on the drugs with positive signals detected, there were 9 antihypertensive drugs. After summarizing according to the random-effects model, the ROR was 2.88 (95% CI, 2.37–3.51). According to the ROR signal intensity, the top three drugs were perindopril [ROR (95% CI), 5.52 (1.63–18.67), PRR (χ^2^), 4.9 (6.0)], nitrendipine [ROR (95% CI), 4.76 (3.53–6.42), PRR (χ^2^), 4.31 (124.88)], and cilazapril [ROR (95% CI), 3.33 (2.05–5.4), PRR (χ^2^), 3.13 (24.69)]. Other antihypertensive agents that detected positive signals were reserpine, nitrendipine, cilazapril, rilmenidine, nimodipine, nicardipine, perindopril, trandolapril, and benidipine, and the other 3 compound preparations were aliskiren/hydrochlorothiazide, amlodipine/valsartan, and valsartan/hydrochlorothiazide. The signal values of all drugs are shown in Supplementary Table S1 and Figure 5G.

## 4 Discussion

In the present study, we comprehensively and systematically analyzed the adverse effects of falls among older patients since the establishment of the FAERS in 2004. According to the ROR and PPR, a total of 258 drugs were detected with a positive signal of causing falls in older individuals. Drugs with positive signals were mainly used. To the best of our knowledge, this is the first study that mined the risk of drug-induced falls in older individuals based on the FAERS database and provided evidence for reducing the risk of falls in older individuals and rational drug use.

Many studies have already analyzed drug factors for falls in older individuals (Hart et al., 2020). The EUGMS task and Finish’s group on fall-risk-increasing drugs found that cardiovascular (de Vries et al., 2018), psychiatric (Seppala et al., 2018a), and other drugs (Seppala et al., 2018b) can increase the risk of falls by an evidence-based evaluation. Woolcott et al. (Woolcott et al., 2009) conducted a meta-analysis of the increased risk of falls among older individuals with nine kinds of drugs and found that the use of sedatives and hypnotics, antidepressants, and benzodiazepines was significantly associated with falls in this population. Moreover, several cohort studies (Rasmussen et al., 2021; Jung et al., 2022) and case‒control studies (Krauss et al., 2005; Najafpour et al., 2019) have analyzed the risk of drug-induced falls. However, these studies all presupposed candidate suspected drugs and then further evaluated the incidence of falling. Consequently, the scope of the drugs of concern is limited, and there is the possibility of missing drugs associated with increased fall risk. Therefore, it is necessary to perform unrestricted risk signal mining based on the FAERS database.

In this study, nervous system drugs were the main drugs with a positive signal detected, accounting for 44.1% of the total drugs with a positive signal. According to the risk signal intensity of various drugs causing falls in older individuals, these drugs included antipsychotics, hypnotics and sedatives, antiparkinsonian drugs, anticonvulsants, central nervous system drugs, and antidepressants, which is consistent with a previously published systematic review (Seppala et al., 2018a). Our results further expanded the scope of the analyses and provided the intensity of risk for specific drugs. The increased risk of falls in older patients caused by antidepressants is related to the adverse effects of these drugs, which mainly include extrapyramidal reactions, orthostatic hypotension, sedative, and anticholinergic effects. In addition, SSRIs may also lead to hyponatremia, and TCA may also be associated with drug-induced syncope *via* Brugada syndrome. The increased risk of falls in older patients caused by antipsychotic drugs is also associated with adverse effects of these drugs, which mainly include extrapyramidal reactions, orthostatic hypotension, sedative, and anticholinergic effects. When the drug needs to be discontinued due to the risk of falls or other reasons, it is usually necessary to gradually reduce the dose, eventually completely stopping the drug, and focus on monitoring fluctuations in symptoms such as anxiety, insomnia, and agitation after drug discontinuation. The increased risk of falls in older individuals treated with antiparkinsonian drugs is associated with hypotension. Previous studies on the risk of falls caused by antiparkinsonian drugs have reported somewhat inconsistent findings. In their meta-analysis, Seppala et al. (Seppala et al., 2018b) reported no significant difference in the impact of antiparkinsonian drugs. In contrast, Bloch et al. (Bloch et al., 2013) found significant differences in the impact of antiparkinsonian drugs. The increased risk of falls in older patients taking benzodiazepine sedative-hypnotic drugs might be related to the sedative, hypnotic, anxiolytic, anticonvulsant, and muscle relaxant effects, where the highest risk of falls occurs within the first 2 weeks of administration. Multiple meta-analyses have shown that benzodiazepines are associated with an increased risk of falls (Woolcott et al., 2009; Seppala et al., 2018a), which is consistent with our results. The increased risk of falls in older patients treated with nonbenzodiazepine sedative-hypnotic drugs may be associated with drowsiness, dizziness, confusion, cognitive impairment, ataxia, and delayed reaction time. Falls are a common cause of injuries and fractures in people with epilepsy. Although some falls may be attributable to seizures, numerous studies have shown that less than half of falls and fractures are directly related to seizures and that falls often occur in patients taking antiepileptic drugs (Leppik et al., 2017). Many case reports have found that treatment with antiepileptic drugs such as phenytoin and valproate or abrupt discontinuation of antiepileptic drugs such as carbamazepine, oxcarbazepine, and valproate may cause reversible splenial lesion syndrome, ischemia and hypoxia of the corpus callosum, or hyponatremia, which in turn can lead to uncoordinated information integration and transmission in the bilateral cerebral hemispheres, thereby increasing the incidence of falls (Maximos et al., 2017).

Antihypertensive drugs are the type of drugs with the highest risk among the nonneurological drugs evaluated in this study, and the effect of antihypertensive drugs on blood pressure is likely to be a key factor leading to falls. However, this is not completely consistent with the previous meta-analysis of cardiovascular drugs (de Vries et al., 2018), which may be due to hypertension, which is extremely prevalent among older individuals. Antihypertensive drugs are widely used in combination with the population reporting adverse effects, which leads to a higher risk based on the FAERS database than in other studies. The American Heart Association noted that for patients with cardiovascular disease, it is especially important to prevent the occurrence of falls (Denfeld et al., 2022), mainly due to the higher risk of falls in this group but also due to the more serious consequences of their falls.

In this study, we performed a comparison of the risk signal intensity of various drugs classified according to pharmacological mechanisms. The higher the ROR value is, the higher the risk of falling in older individuals. When selecting therapeutic drugs in clinical practice, especially for older patients evaluated as having a high risk of falls, it is recommended to give priority to drugs with no positive signal detected for treatment. If these drugs do not meet clinical needs, it is recommended to prioritize drugs with lower risk, i.e., drugs of the same pharmacology with lower ROR values. In addition, targeted prevention, management, and pharmaceutical monitoring should also be performed, especially for polypharmacy requiring special vigilance. Both of the interaction of drugs and the additive risk of multiple drugs used can make patients more likely to falls.

The present study still has some limitations: 1) proportion imbalance analysis is a statistical method used to determine the correlation between the targeted drug and adverse effects; however, it cannot clarify the causal relationship between the targeted drug and ADE and cannot exclude other confounding characteristics, such as age, sex, country, ethnicity, comorbidities, underlying diseases and concomitant medications; 2) as data in the FAERS database are reported spontaneously and voluntarily, which may be affected by recent studies or the media, it may contribute to a certain bias (Montastruc et al., 2006; Maciá-Martínez et al., 2016). 3) Although the simple size in this study is relatively large, data from the other available databases should be included or applied for verification.

## 5 Conclusion

Many drugs were associated with a high risk of falls in older patients. The drug is one of the important and preventable factors for fall control, and the risk level of drug-induced falls should be considered to optimize drug therapy in clinical practice.

## Data Availability

The original contributions presented in the study are included in the article/Supplementary Material, further inquiries can be directed to the corresponding author.
